# Dietary Modulation of Oxidative Stress in Alzheimer’s Disease

**DOI:** 10.3390/ijms18071583

**Published:** 2017-07-21

**Authors:** Arjun Thapa, Nick J. Carroll

**Affiliations:** Department of Chemical and Biological Engineering and the Center for Biomedical Engineering, University of New Mexico, Albuquerque, NM 87131, USA

**Keywords:** oxidative stress, pro-oxidant, degenerative disease, Alzheimer’s disease, diet, anti-oxidant, flavonoid, polyphenol, protective function

## Abstract

Cells generate unpaired electrons, typically via oxygen- or nitrogen-based by-products during normal cellular respiration and under stressed situations. These pro-oxidant molecules are highly unstable and may oxidize surrounding cellular macromolecules. Under normal conditions, the reactive oxygen or nitrogen species can be beneficial to cell survival and function by destroying and degrading pathogens or antigens. However, excessive generation and accumulation of the reactive pro-oxidant species over time can damage proteins, lipids, carbohydrates, and nucleic acids. Over time, this oxidative stress can contribute to a range of aging-related degenerative diseases such as cancer, diabetes, macular degeneration, and Alzheimer’s, and Parkinson’s diseases. It is well accepted that natural compounds, including vitamins A, C, and E, β-carotene, and minerals found in fruits and vegetables are powerful anti-oxidants that offer health benefits against several different oxidative stress induced degenerative diseases, including Alzheimer’s disease (AD). There is increasing interest in developing anti-oxidative therapeutics to prevent AD. There are contradictory and inconsistent reports on the possible benefits of anti-oxidative supplements; however, fruits and vegetables enriched with multiple anti-oxidants (e.g., flavonoids and polyphenols) and minerals may be highly effective in attenuating the harmful effects of oxidative stress. As the physiological activation of either protective or destructive pro-oxidant behavior remains relatively unclear, it is not straightforward to relate the efficacy of dietary anti-oxidants in disease prevention. Here, we review oxidative stress mediated toxicity associated with AD and highlight the modulatory roles of natural dietary anti-oxidants in preventing AD.

## 1. Oxidative Stress and Aging Related Diseases

Cells gradually degenerate during aging. Aging is a highly complex process because cellular degeneration involves thousands of different mechanisms and processes [1,2,3,4]. During aging, cellular metabolic redox reactions induce harmful genetic and biochemical alterations [1,5,6,7,8]. An uncontrolled production of reactive oxygen and nitrogen species damages cellular proteins, lipids, carbohydrates, and nucleic acids through oxidative stress, contributing to cell degeneration during aging [3,5,9,10,11,12,13]. Reactive oxygen species (ROS) and reactive nitrogen species (RNS) are short-lived, but uncontrolled redox reactions continuously generate new ROS and RNS, and are believed to contribute to the onset of various degenerative diseases including cancer, diabetes, age-related macular degenerations, and Alzheimer’s, and Parkinson’s diseases [1,5,8,14,15,16].

Metabolically active cells and tissues have high demand for oxygen and generate substantial amounts of ROS [1,17,18]. Metabolic and physiological behavior is known to change rapidly with increasing aging. Thus, high performance cells and tissues, including post-mitotic cells (e.g., brain and heart cells) are most likely to be severely affected by ROS overload. This is one of the fundamental bases for the hypothesis of oxidative stress induced degenerative diseases among elderly individuals. The original theory on oxidative stress and aging was first proposed by Herman in 1956 [19]. It was suggested that the unpaired electrons: (i) mediate free radical reactions; (ii) generate ROS and RNS by-products during aerobic respiration; and (iii) exert damaging modification of cellular components. Hydroxyl (OH^•^), superoxide (O_2_^•^^−^), nitric oxide (NO^•^), nitrogen dioxide (NO_2_^•^), peroxyl (ROO^•^), and lipid peroxyl (LOO^•^) are the most common ROS and RNS molecules associated with aging related degenerations [8,20]. Other oxygen and nitrogenous by-products such as hydrogen peroxide (H_2_O_2_), ozone (O_3_), singlet oxygen (1O_2_), hypochlorous acid (HOCl), nitrous acid (HNO_2_), and peroxynitrite (ONOO^•^) are mediators of free radical reactions [1,8,20]. Both enzymatic and non-enzymatic cellular reactions generate ROS and RNS molecules. The mitochondrial respiratory chain and the phagocytosis system generate ROS and RNS enzymatically [1,9]. Ionic radiation and smoking are non-enzymatic generators of free radicals [21,22]. The sources of ROS and RNS molecules can be exogenous or endogenous. Inflammation, stress, infection, and excessive exercise serve as endogenous source of the ROS and RNS, whereas smoking, drinking, heavy metals, and radiation are canonical exogenous sources [6,9,23].

Pro-oxidant ROS and RNS molecules are continuously produced in the cells throughout life. Mitochondria, the “powerhouses” of cells, are the main source of ROS and RNS production. For example, the oxidation of the reduced form of nicotinamide-adenine dinucleotide (NADPH) in the mitochondrial membrane involves a four-electron reduction of O_2_ to H_2_O and releases energy [1,9,24]. The released energy is utilized to phosphorylate ADP to ATP. One electron reduction of O_2_ to superoxide (O_2_^•^^−^) also takes place in the mitochondrial electron transport chain [1,9,24]. These superoxide anions (O_2_^•^^−^) are consumed by mitochondrial manganese superoxide dismutase (SOD) and hydrogen peroxides (H_2_O_2_) are produced [1,9,25,26]. H_2_O_2_ is either converted into water by catalase or harmful hydroxyl radicals (OH^•^) by reduced transition metals. H_2_O_2_ generated by mitochondrial peroxysomal β-oxidation of fatty acids is another source of free radicals within the mitochondria [1,9]. Moreover, phagocytosis processes generate significant amounts of ROS in cells. Pro-inflammatory cytokines, such as interluekin-1 and interferon-γ also produce large amounts of reactive nitric oxide by inducing nitric oxide synthase (iNOS) [6,24,27,28,29].

## 2. Oxidative Stress and Alzheimer’s Disease

Normal metabolic processes or cellular stresses continuously generate ROS and RNS and they accumulate over time [1,7,16,30,31]. The overexposure to ROS, and its cumulative effects, result in cellular degeneration and impaired regeneration and are fundamentally associated with aging related degenerative diseases. Increased levels of pro-oxidant impairment and a weakening in anti-oxidative defense mechanisms are most common in elders, suggesting that the elderly population is most affected by associated devastating degenerative diseases. Below, we will focus on oxidative stress induced mechanisms, which are potentially linked to aging related Alzheimer’s disease (AD).

AD is a complex and chronic aging related neurodegenerative disorder [3,4,32,33,34]. The mechanisms of AD are largely unknown. Inflammation, intracellular calcium ion release, autophagy, apoptosis, and, importantly, over production and aggregation (crosslinking) of Aβ peptides are widely believed to be involved in AD neurodegeneration [6,29,32,33,35,36]. Neuronal cells, among others, are particularly susceptible to oxidative stress induced degenerations [17,18]. Neurons are metabolically active cells and utilize large amounts of oxygen, roughly one fourth of the total oxygen consumed in the body, during metabolism. As a result, neuronal cells generate tremendous amount of ROS and RNS, and are subject to free radical attacks. Compared to other cells, neurons comprise a low level of anti-oxidant defense molecules such as glutathione (GSH), and contain a higher amount of oxidation prone polyunsaturated fatty acids [1,29,37]. The presence of anti-oxidative enzymes, hemeoxygenase (OH)-1 and superoxide dismutase (SOD)-1 in senile plaques clearly indicate the involvement of oxidative stress in AD pathology [1,29,38]. Importantly, oxidative stress has been reported to be a relatively early event in AD pathogenesis [39].

Enhanced ROS generation and accumulation increase the levels of oxidized proteins, lipids, and DNA; these oxidized species have been implicated in AD [12,14,29,40,41,42]. Amyloid-β (Aβ) peptide deposition in the brain is a hallmark of AD [34,42]. ROS molecules have been shown to enhance Aβ generation, misfolding, and aggregation (crosslinking) [39,43]. The predominant Aβ peptide isoforms comprise 40–42 amino acids chains (sequence: DAEFRHDSGYEVHHQKLVFFAEDVGSNKGAIIGLMVGGVVIA) [33,34,43]. Aβ peptides are proteolytic products of amyloid precursor protein (APP). Genetic mutation(s) in the APP gene makes it susceptible to be cleaved by β- and γ-proteases and generate Aβ peptides [33,34]. Methionine (at position 35, underlined above in the Aβ protein sequence) in the Aβ peptide is a highly oxidizable amino acid residue [41,42,44,45]. Side chains of methionine are oxidized by ROS at physiological conditions, and oxidized forms of methionine are present in AD brain tissue. Methionine may undergo a two-electron oxidation to methionine sulfoxide or one electron oxidation to a sulfuranyl free radical, resulting in induced damage to surrounding neuronal proteins and lipids [41,43,45].

Lipid oxidation is one of the pathological markers found in AD brain tissue [13,38,42,43]. Highly oxidizable polysaturated fatty acids such as arachidonic and docosohexanoic acids are present in the brain [42,45]. Lipid peroxidation damages neuronal membranes and generates a number of secondary products, including 4-hydroxy-2-nonenal, acrolein, isoprostanes, and neuroprostanes [12,42,43,46]. One study measured the lipid peroxidation biomarker (non specific) thiobarbituric acid reactive substances (TBARS) and found higher levels of TBARS in the synaptosomal membrane fraction of the AD neurons [38].

In addition to Aβ oxidation and lipid peroxidation, oxidative DNA damage is also implicated in AD, whereby ROS damage DNA by unregulated modification of nucleic acids bases [1,13,47,48,49]. For instance, hydroxyl radicals can modify guanine nucleotides to form 8-hydroxy guanine [13,47,49]. The 8-hydroxy guanine species preferably base pairs with adenine instead of cytosine. Thiamine oxidation can generate base pairing between 5-hydroxymethyluracil and adenine [13,47,49]. Furthermore, RNS and peroxynitrous acid react with nucleic acid bases to cause oxidative deamination by replacing NH2 with OH groups. Moreover, adenine, cytosine, and guanine are converted into hypoxanthine, uracil, and xanthine, respectively, by deregulated oxidative species. Hypoxanthine and uracil can mispair with cytosine and adenine, respectively. The detection of oxidized DNA bases, namely, 8-hydroxyadenine, 8-hydroxyguanine, thymine glycol, Fapy-guanine, 5-hydroxyluracil, and Fapy-adenine, in an AD brain clearly implicates the crucial role of DNA damage in AD pathogenesis [13,47,48,49]. Studies have also revealed significantly higher amounts of nicked DNA from neurons exposed to Aβ peptides [48] in AD patients.

As many as one dozen metals, including copper, iron, aluminum, lead, mercury, manganese, and zinc ions, have been reported to help maintain normal cellular homeostasis [33]. However, elevated levels of heavy metals have been reported in the brain tissue of AD patients [29,33,45]. Several reports have shown that the interactions of metal ions with APP and Aβ peptides enhance ROS formation, leading to Aβ over-production, aggregation and enhanced neurotoxicity [29,39,40]. Heavy metal ions function as potent pro-oxidants and mediate redox reactions that form highly reactive free radicals such as hydroxyl ions. Lead and mercury have been reported to enhance the expression of APP, glial cell reactivity, neuronal inflammation, and oxidative stress.

## 3. Beneficial Effects of Oxidative Stress in Disease Resistance

Several decades after the original theory of oxidative stress and aging [19], our understanding of oxidative stress has advanced significantly. Although a large body of evidence univocally reveals the potential roles of oxidative stress in cellular degenerations linked to a wide-ranging aging related degenerative diseases, recent studies show the important roles of oxidative stress in immune defense, molecular signaling, and cell survival [7,16,24,30,50]. Phagocytic cells, neutrophils, macrophages, and monocytes utilize free radicals to destroy invading pathogens. Given that ROS and RNS have short half-lives, and ROS and RNS modified proteins and lipids perform redox signaling and transcription factor activation, oxidative stress is not always harmful.

Nitric oxide is an intracellular messenger involved in modulating blood flow, clotting, and neuronal activity [51]. Increasing evidence shows the beneficial roles of protein and lipid oxidation products. Oxidative stress inducers such as hypoxia, hyperoxia, hypothermia, hyperthermia, ischemia, and mitochondrial electron transport inhibitors perform adaptive cellular functions [16]. Okabe et al. showed the important role of the endogenous oxidative stress inducer 24-*S*-hydroxycholesterol in brain cholesterol homeostasis using a neuroblastoma cell model [52]. Methionine oxidation of Aβ peptide has been described to reduce Aβ aggregation, alter Aβ conformations, and attenuate Aβ toxicity [53]. Singlet oxygen generation has been suggested to offer beneficial roles in taupathies [13]. Protective functions of cysteine’s sulfhydryl group (-SH) to sulfenation (-SOH) conversion in DJ-1 protein (protein associated with Parkinson’s disease) have been reported [7]. Another study on α-synuclein showed methionine oxidation generates synuclein protein aggregates that are non-toxic to primary cells [54]. It is possible that in these situations, ROS is transiently generated at moderated levels and the basal levels of ROS offer cellular defense and enhance cell survival.

## 4. Anti-Oxidative Defense in Alzheimer’s Disease

Anti-oxidants are key constituents that neutralize pro-oxidant molecules, mediate redox reactions, and attenuate oxidative stress [1,31,55]. Under normal conditions, cells are well equipped with anti-oxidant defense mechanisms to resist against oxidative stresses [7,16,30,31,55], thereby maintaining safe levels of free radicals. By contrast, higher levels of ROS and other free radicals (e.g., superoxide anions, hydrogen peroxide, hydroxyl, peroxyl, singlet oxygen, and nitric oxide) are present in the brain tissue of AD patients, indicating the crucial role of ROS and RNS in AD pathology [13,38,40,56]. It has been reported that AD patients have low levels of pro-oxidant defending, anti-oxidative GSH molecules in their brain, blood, and cerebrospinal fluid [38]. Compared to age matched, healthy individuals, AD patients have a decreased ratio of reduced and oxidized levels of GSH [13,37]. Reduced levels of endogenous anti-oxidants, α-tocopherol (vitamin E), and ascorbic acid (vitamin C) have also been shown in the plasma and brains of AD patients [13,37,57]. These key anti-oxidant defense molecules help prevent lipid peroxidation. Moreover, reduced levels of the essential trace element selenium and enzymes containing selenocysteine residues that catalyze thioredoxin-1 (oxidative stress regulating protein) have been documented in AD [13,58]. Serum analyses of AD patients also show a decreased level of SOD (catalytic enzyme converting superoxide to peroxide) [13,59]. These findings suggest weakened anti-oxidant defense mechanisms may contribute to ROS overload in AD. Older people are thus at greater risk for AD pathogenesis, as weakened anti-oxidant defenses (due to reduction in SOD and GSH levels) and concomitant increase in ROS levels commonly occur as aging progresses [59,60].

## 5. Natural Anti-Oxidative Therapeutics in Alzheimer’s Disease

**Figure 1:**
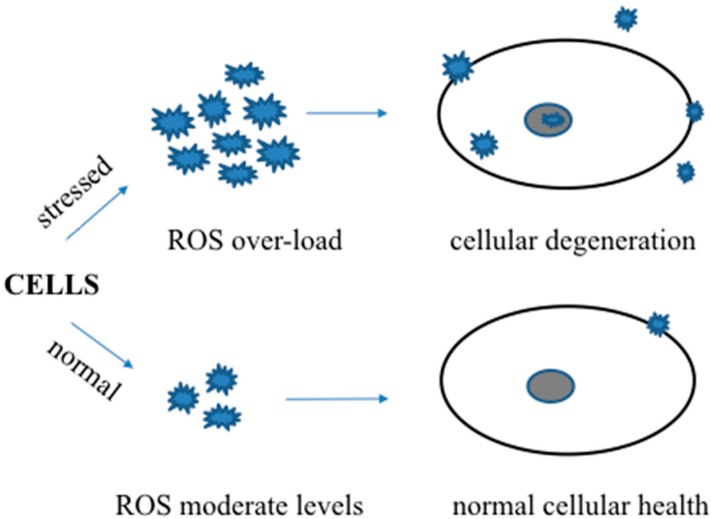
A schematic showing the beneficial and harmful roles of reactive oxygen or nitrogen species in normal and stressed conditions.

It is now evident that pro-oxidant overload is critically associated with degenerative diseases connected to advanced aging (Figure 1) and that anti-oxidant defense mechanisms could play vital roles to attenuate the activity of pro-oxidants to help prevent disease progression. Promising agents include SOD, catalase, and glutathione peroxidase, which are some of the primary cellular enzymes that scavenge ROS [1,3,13,45]. Glutathione reductase and glucose-6 phosphate dehydrogenase enzymes maintain the regular supply of GSH and NADPH for optimum functioning of primary anti-oxidant defense enzymes. Other anti-oxidant molecules such as vitamins A, C, and E, β-carotene, etc., are also involved in regulating cellular ROS levels [1,13,61,62,63]. ROS over-production and accumulation inhibits anti-oxidative stress defense mechanisms. Thus, boosting anti-oxidant pathways and/or neutralizing pro-oxidants by exogenous anti-oxidants may provide viable preventive options for degenerative diseases.

**Figure 2:**
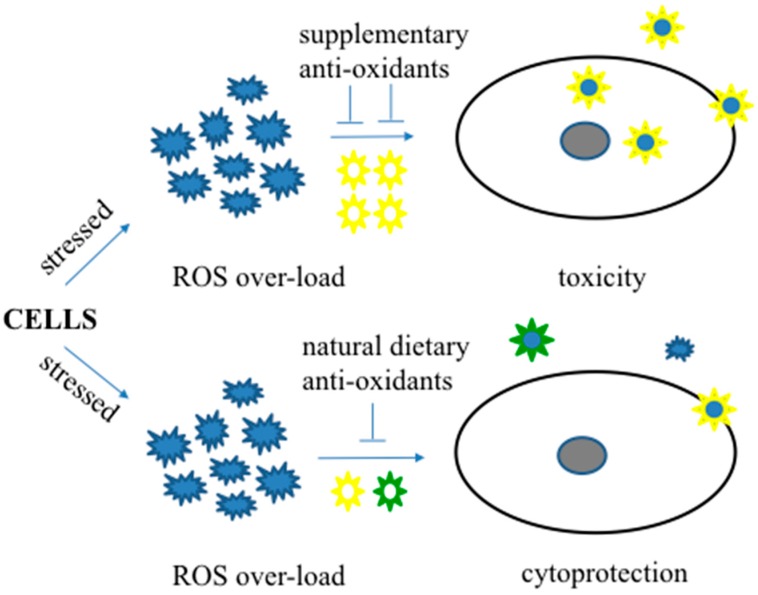
A schematic showing the beneficial roles of dietary anti-oxidants (moderate amounts of multiple anti-oxidants) and harmful roles of anti-oxidant supplements (higher amounts of individual anti-oxidant) during stressed conditions.

Several different kinds of anti-oxidative agents, acting alone or in synergistic combinations, have been suggested to prevent AD, but the outcomes of current anti-oxidant therapeutics are inadequate. Recent studies have shown that higher doses of anti-oxidative formulations are ineffective and also have negative side effects [13,62,64,65]. Considering the protective roles of moderate levels of oxidative stress [7,16,24,30], anti-oxidative therapeutics that attenuate the harmful activity of deregulated pro-oxidants while allowing cells to maintain moderated levels of native pro-oxidants may offer necessary beneficial effects (Figure 2). Natural products (fruits and vegetables) are rich in diverse anti-oxidative components, including vitamins and essential minerals. Such balanced dietary formulas may play a beneficial role to prevent AD. Although the mode of action and targets of the dietary anti-oxidants remains unclear, multiple anti-oxidative species found in fruits and vegetables have been shown to activate a multitude of cell protective pharmacological pathways and offer beneficial roles in mitigating age-related pathologies.

Flavonoids and polyphenols are major anti-oxidative molecules found in fruits and vegetables. Flavonoids are C6-C3-C6 backboned compounds and consist of two aromatic rings (α and β) linked with a pyran ring (γ) [29,66,67]. Based on the oxidation state of the pyran ring, flavonoids are classified into flavones, flavonols, flavanols, isoflavonoids, flavanones, and anthocyanins. Flavones (e.g., apigenin and luteolin) are flavonoids enriched within in parsley and celery. Flavonols are keto-hydroxypyrene containing flavonoids (e.g., myricetin and quercetin) present in onions and broccoli. Flavanols consist of a hydroxylatedpyran ring at the C3 position. These compounds are rich in green tea and red wine. Genistein and daidzein found in soy products are isoflavonoids, which have a substituted keto group on a pyran ring structure. The flavanone flavonoids (e.g., hesperetin and naringenin) are abundant in citrus and tomatoes. These consist of substituted keto-pyran groups on the α ring at the C3 and C5 position. Anthocyanins are colorful pigments, which are rich in apples and berries. Phenolic acids (e.g., caffeic acid and gallic acid), lignans (e.g., secoisolariciresinol), stilbenes (e.g., resveratrol), and curcuminoids (e.g., curcumin) are non-flavonoid types of polyphenols [29,67].

Flavonoids and polyphenols are potent dietary scavengers of pro-oxidants and offer protective functions against oxidative stress induced diseases by neutralizing destructive free radical reactions and/or quenching reactive metals that generate ROS [16,68,69,70]. These scavengers also reduce peroxide concentrations and repair oxidized membranes, enhance the activation of anti-oxidant enzymes, and increase anti-oxidant levels. As varieties of flavonoids and polyphenols are found in vegetables and fruits, a daily healthy diet is highly attractive as a potential preventive therapeutic approach against degenerative diseases. Curcumin is a powerful anti-oxidative polyphenol known for more than a thousand years as a beneficial agent in dozens of aging related degenerative diseases, including AD [71,72,73]. The two terminal phenyl rings of curcumin serve as free radical scavengers and terminate harmful redox reaction chains. Furthermore, curcumin chelates heavy metal ions such as Fe^2+^ and attenuates their oxidizing capability, thereby reducing oxidative stress by reducing free radical levels [72]. In vitro anti-oxidant activity analysis of curcumin shows that it can efficiently scavenge 1,1-diphenyl-2-picryl-hydrazyl (DPPH^•^), *N*,*N*-dimethyl-*p*-phenylenediaminedihydrochloride (DMPD), 2,2′-azino-bis(3-ethylbenzthiazoline-6-sulfonic acid) (ATBS), superoxide anion and hydrogen peroxide [28]. There are also reports that curcumin increases cellular expression of hemeoxygenase, GSH, catalase and erythroid 2 p45 (NF-E2)-related factor (Nrf2) [67], which are the important regulatory molecules associated with anti-oxidant defense mechanisms.

*Ginkgo biloba* extract (EGb 761) has been shown to be effective in various aging related diseases [74,75]. Flavonoids and terpens present in ginkgo ameliorate mitochondrial respiratory chain function by quenching superoxide anion, hydroxyl, and peroxyl free radicals [13,76]. *Ginkgo biloba* facilitates neurotransmitter uptake and prevents oxidative stress induced neuronal apoptosis. It also inhibits TBARS (lipid peroxidation marker) induced by *t*-butyl hydroperoxide [77]. The protective effects of *Ginkgo biloba* correlate and the associated reduction of toxic Aβ aggregates was shown to improve cognitive functions in mice [74,75].

Vitamins C and E, and carotenoids are other well-known anti-oxidants [55,63,78]. Humans are unable synthesize these vital molecules; thus, strategic dietary sources are vital. Vitamin C is a potent electron donor and serves as the first defense against free radicals in blood and plasma. It is a powerful inhibitor of lipid peroxidation and facilitates vitamin E synthesis in the lipoproteins and membranes. It also diminishes α-tocopheroxyl free radical propagations in membranes and regenerates α-tocopherol (vitamin E) [78]. Vitamin E protects against oxidative damage by ROS through the inhibition of oxidative modification of low-density lipoproteins [79]. It has been to shown to reduce isoprostanes (biomarkers for lipid peroxidation) levels in animal models. The formation of isoprostanes increases significantly in animals deficient in vitamin E [80]. Vitamins C and E have shown to synergistically interact to offer protection against lipid peroxidation. In AD rat models, vitamin E suppressed brain lipid peroxidation and Aβ-levels [81].

## 6. Limitations of Anti-Oxidative Therapeutics in Alzheimer’s Disease

Growing evidence suggests that naturally available anti-oxidants are promising small molecules in preventing AD and other degenerative diseases. To date, flavonoids and polyphenols such as curcumin, *Ginkgo biloba*, and vitamins C and E have been tested for different aging related clinical trials [13,61,62,73,74,76]. Considering the strong anti-oxidative and cognitive enhancing capability of curcumin [82,83], it was taken to clinical trials for cancer, diabetes, and AD. The results of the curcumin trial were discouraging, ostensibly due to its poor uptake in the gastrointestinal tract. *Ginkgo biloba* also did not show positive therapeutic effects in a large randomized control trial [13,76]. A study by Lloret et al. shows vitamin E lowers oxidative stress in some AD patients [84]. However, lipophilic vitamin E partitions into membranes and oxidized vitamin E can accumulate in the membranes or pass the electrons to another lipid, which may further damage the membrane [85].

There are a number of limitations regarding the analysis of clinical trials that explore the effects of anti-oxidants in AD [13,61,62]; (i) subjects who are already diagnosed with the clinical conditions are included; (ii) elderly persons who are taking other prescription pills are also involved; and (iii) reliable biochemical markers (for detecting pro- and anti-oxidants) in animal models are not available. Trial failures clearly suggest a more detailed study on the mechanism of oxidative stress and aging is needed, and elucidation of the relationship between pro- and anti-oxidant molecules is necessary for informing trial paradigms. It is also possible that anti-oxidants consistently administered years before pathogenesis may offer mitigating effects. Anti-oxidants are often tested individually, but synergistic combinations of anti-oxidants may be more effective. For example, studies have shown that vitamin C and GSH anti-oxidants are interdependent for their optimal anti-oxidative performance [13]. Another critical consideration should be given to the anti-oxidant dose. Supplementary vitamins and trace elements can lead to harmful conditions at higher doses [13,61]. Anti-oxidant efficiency may also depend on the timing of the consumption. It was found that vitamins C and E should not be taken during daytime hours [86]. Hence, the relevance of circadian rhythm principles may also be key considerations.

## 7. Dietary Approaches to Anti-Oxidative Therapeutics in Alzheimer’s Disease

Increasing evidence shows that hundreds of different flavonoids and polyphenols potently scavenge ROS and RNS and play beneficial roles in aging related degenerative diseases [16,28,29,50,67]. However, some of the tested anti-oxidant formulations have not shown expected positive effects on clinical trials [13,62,65,83,87,88]. Therapeutic targeting against degenerative diseases is a challenging task because the molecular mechanisms of these diseases are complex and largely unknown. Degenerative diseases, including AD, are influenced by hundreds of genetic and environmental factors, and progress via a number of different mechanisms [34,35,37,42,89].

Since ancient times, diets rich in polyphenol and flavonoid anti-oxidants have been known to offer health benefits against aging related diseases. Recent studies point out that the natural compounds with multiple polyphenol and flavonoid groups may be a better protective agent against such diseases. Enhanced anti-oxidant activity, possibly due to presence of more hydroxyl groups, and cytoprotective effects of bi-flavonoids compared to flavonoid monomers [29,68,90], have been shown to play beneficial roles in wide-ranging degenerative diseases such as AD and cancers. Bi-flavonoids are also endowed with multiple capabilities such as metal chelating, inhibiting protein oxidation and lipid peroxidation [29,90,91,92]. Dimers of apigenin revealed enhanced anti-cancer effects compared to apigenin (monomeric flavonoid) alone [91,93]. In vitro studies show the protective effects of multiple flavonoid (e.g., bi-flavonoids) and polyphenol (e.g., tannins) containing compounds in AD [66,89,90,94,95]. However, larger flavonoids or polyphenols may have poor blood brain permeability. It is also possible that in vivo degradation of products of these large molecules may function as individual flavonoid or polyphenol moiety [94,95]. Phenolic groups found in curcumin as individual, ferulic acid, and styryl benzene are also strong anti-oxidants [96,97]. These suggest multiple polyphenol and flavonoid groups containing natural anti-oxidants may be better and more efficient anti-oxidant molecules. Because supplementary anti-oxidant formulations do not show promising beneficial effects [64,85,88,98], the consumption of natural dietary compounds may be the best currently available option to prevent degenerative diseases. A variety of fruits and vegetables that are rich in flavonoid and polyphenol content and type may be useful in retarding or reversing the multi-stage pathological events associated with aging and oxidative stress, and clearly suggests a healthy diet plays a positive modulatory role in preventing such diseases.

## 8. Conclusions

Structural damage and the associated deregulation of protein, lipid, and DNA homeostasis are oxidative stress induced events/cascades linked to many degenerative diseases [1,10,39,49,99]. Oxidative stress induced over-production of altered proteins (e.g., Aβ peptides in AD), aggregation (cross-linking), oxidation and DNA [49] and membrane alterations are found in wide-ranging degenerative diseases, and suggests a central role of oxidative stress in the pathogenesis of degenerating diseases, including AD [12,14,40,44,49,100,101]. Therefore, anti-oxidants that neutralize the effects of oxidative stress are promising therapeutics to prevent AD and various degenerative diseases. However, the therapeutic outcomes of the current anti-oxidant formulations against these ailments are discouraging. It is possible that dietary dosage formulations even consume the moderate levels of pro-oxidants that are necessary for maintaining healthy cellular defense and signaling.

Natural fruit and vegetable products that are rich in anti-oxidants (flavonoids and polyphenols) are known as beneficial agents against aging-related disease and improving cognitive functions [29,50,67,69,82,102,103,104]. Therefore, dietary compounds are promising therapeutics for AD and other aging related degenerative diseases. However, currently available dietary supplementary formations have been ineffective in clinical trials as proposed. In fact, some of them even revealed negative side effects [64,65,85,88,98,105,106]. The benefits of several different kinds of natural compounds present in our balanced diet may potentially outweigh the supplementary risks. Fruits and vegetables are enriched with moderate amounts of multiple anti-oxidants and other essential elements. For example, one apple contains quercetin, kaempferol, myricitin, catechin, gallic acid, phloridzin, chlorogenic acid, procyanidin B2 molecules, minerals (calcium, magnesium, phosphorus, and iron), and fiber [95,107]. The individual or synergistic effects of varieties of compounds found in fruits and vegetables could play a transformative role in attenuating oxidative stress. Compounds such as highly hydroxylated polymeric polyphenols and flavonoids, and anthocyanins found in apples and berries, either as a whole or as a modified product, may serve as efficient anti-oxidants [6,29,94,95,107]. Minor active chemical components of the natural product, as has been shown in curcuminoids case [108], may be as effective as major ones.

In conclusion, moderate amounts of multiple anti-oxidants found in a healthy natural diet may efficiently attenuate free radical attacks and neutralize deregulated pro-oxidants while maintaining necessary levels of essential pro-oxidants in the cellular system, with the potential to mitigate oxidative stress induced degenerative diseases, including AD. Because oxidative stress can start early in life, preemptive dietary intervention may be highly beneficial in preventing the progression of degenerative diseases.
